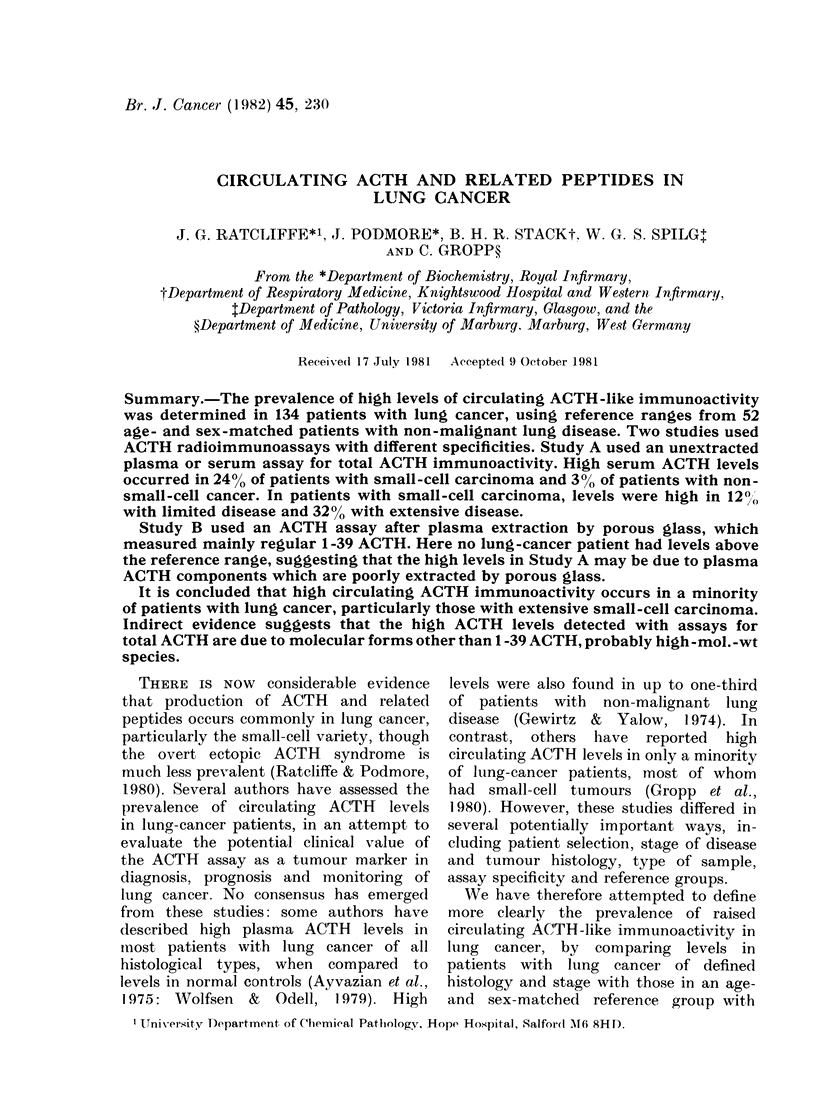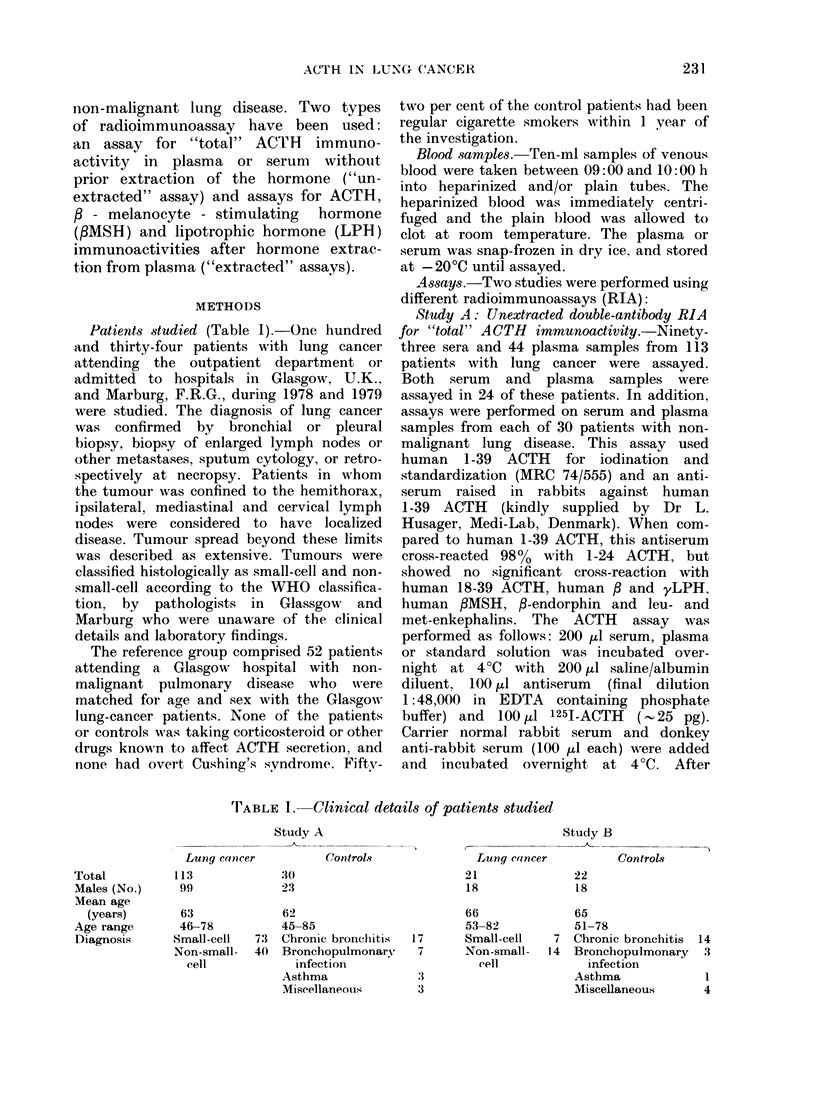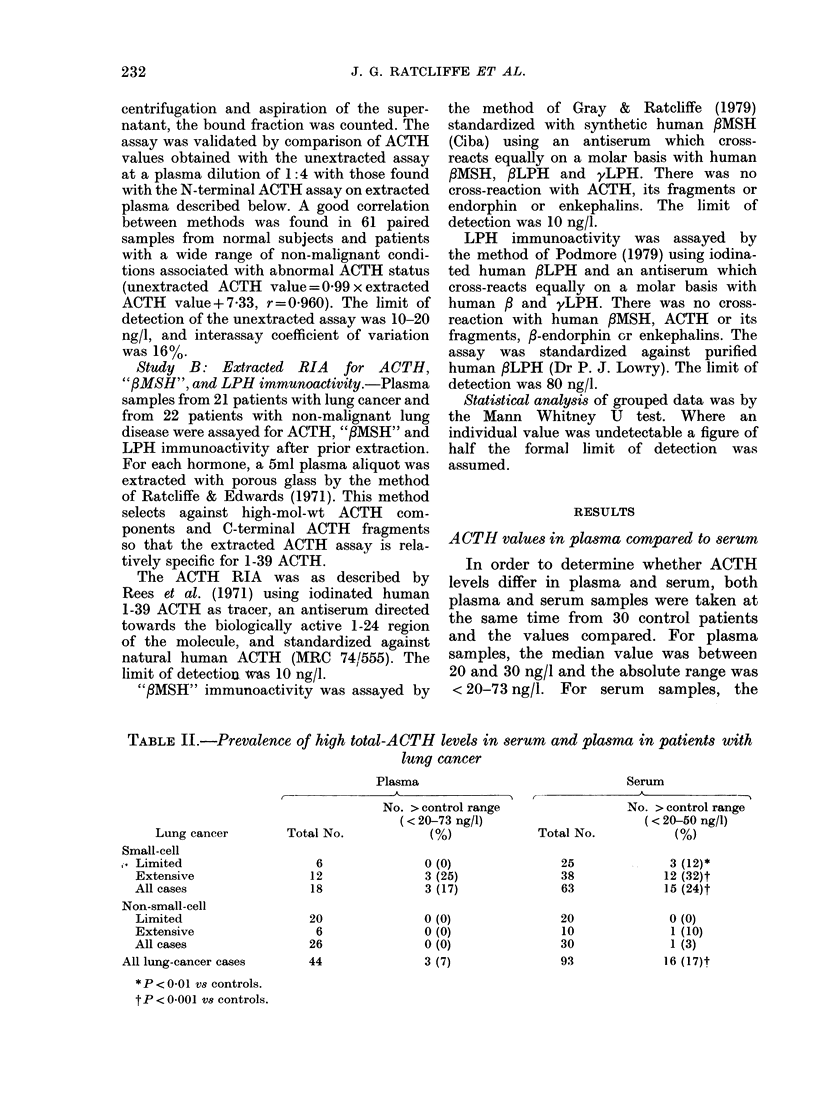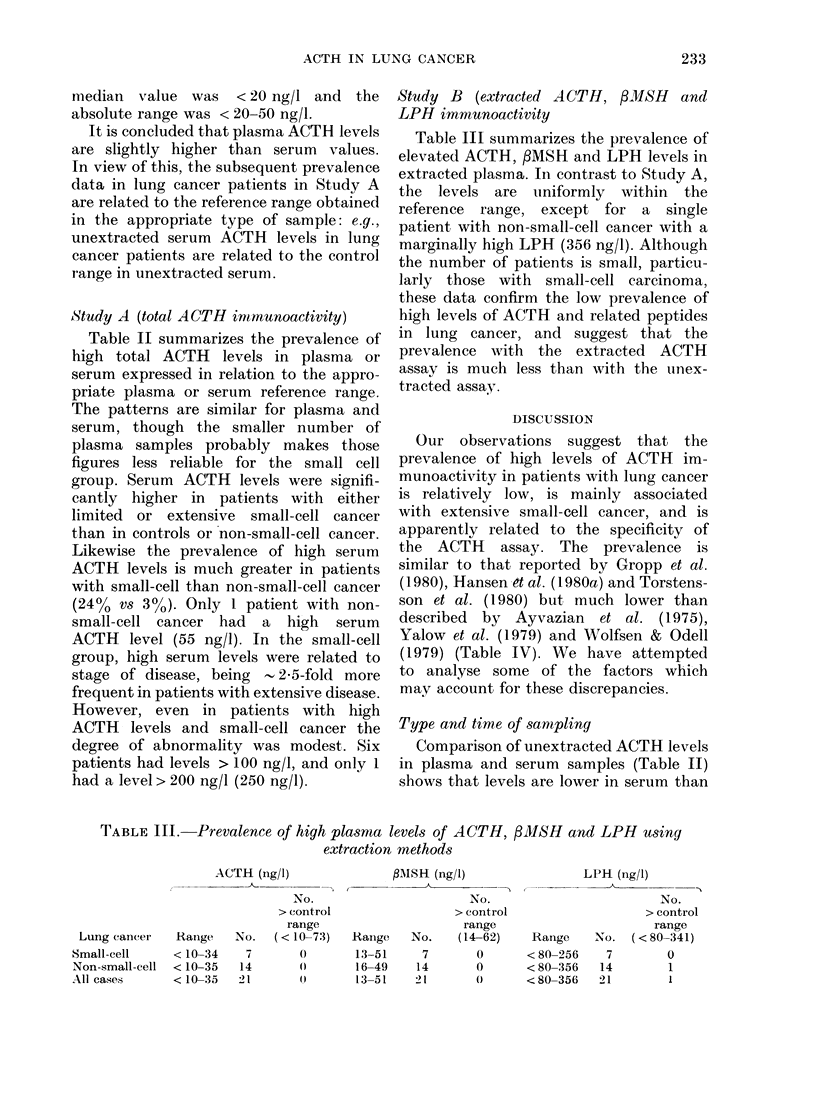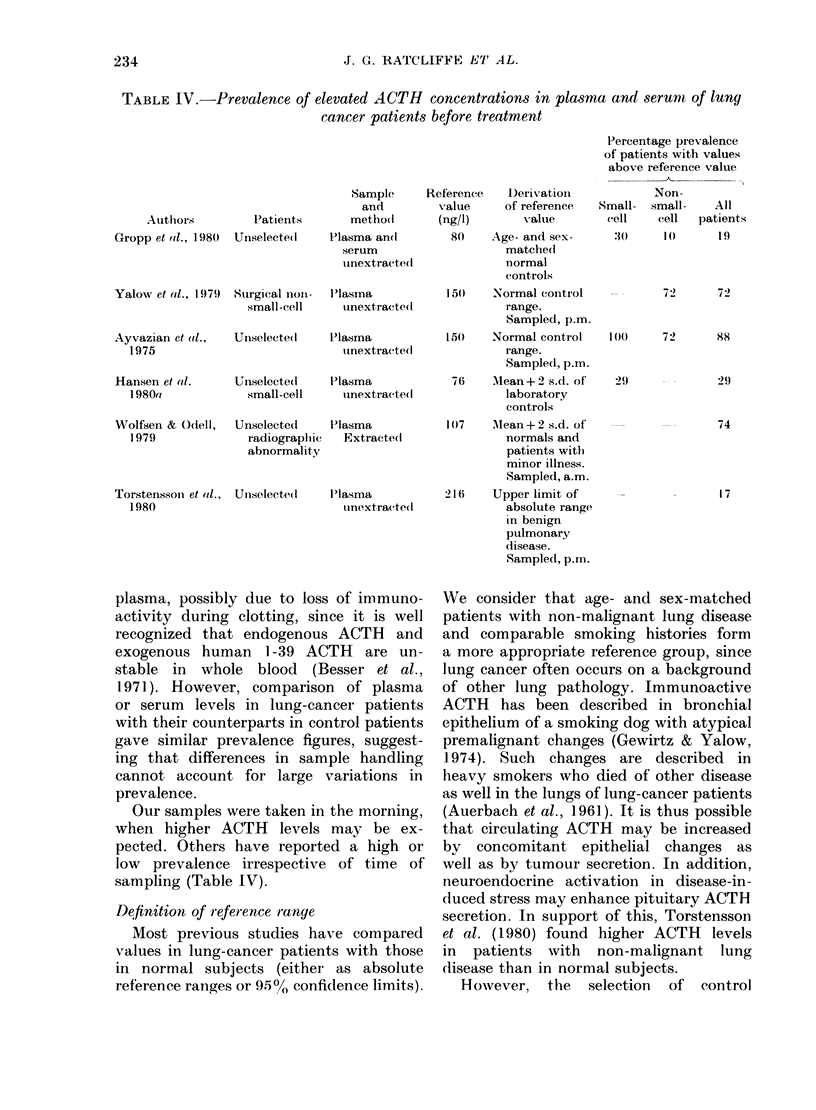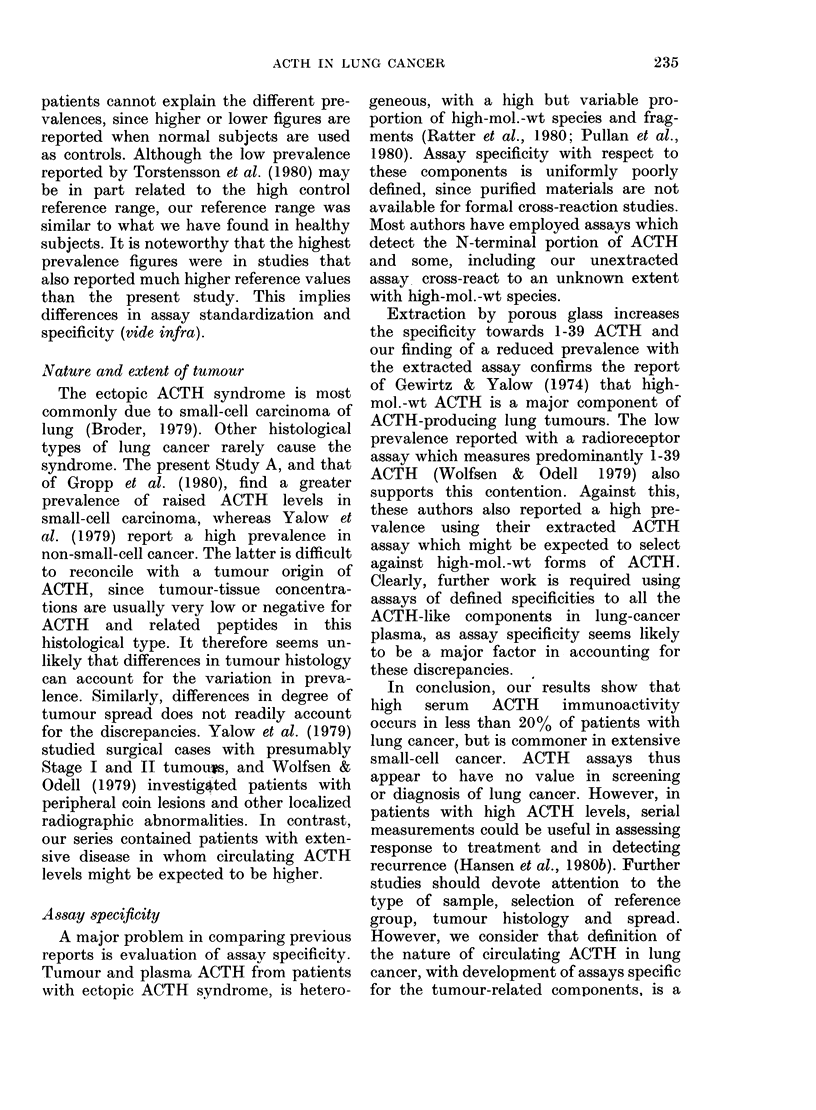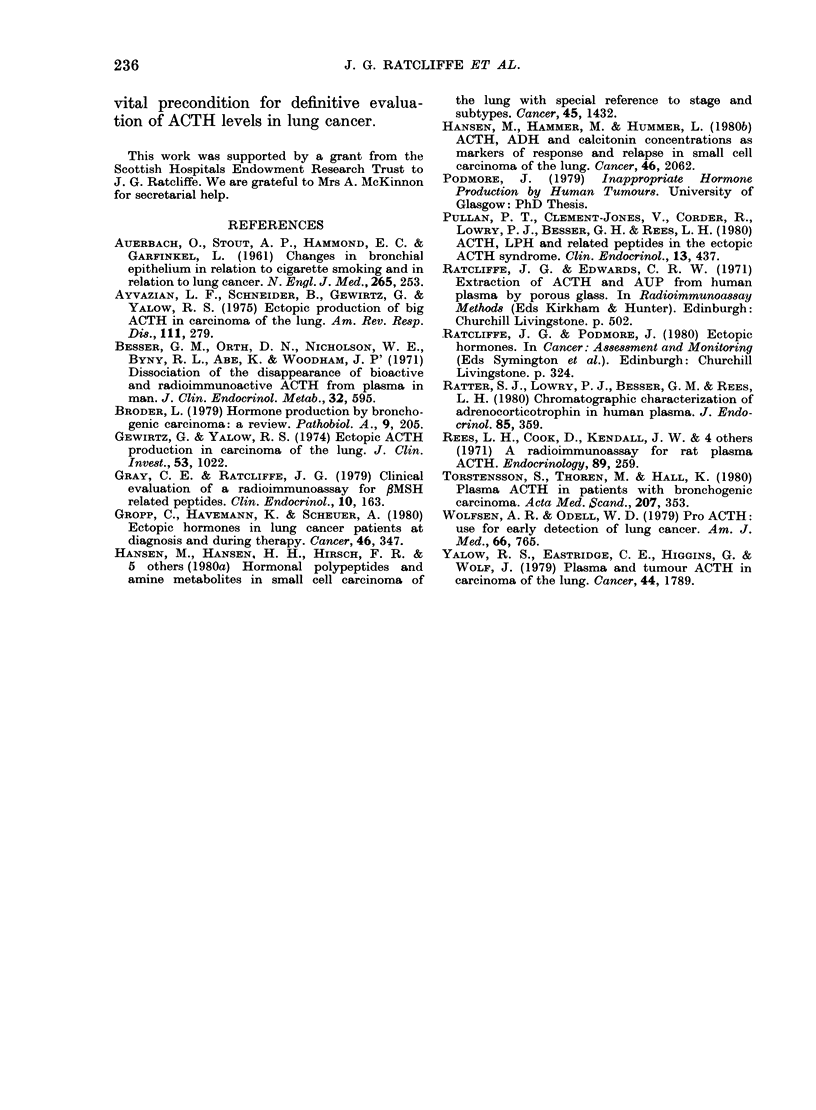# Circulating ACTH and related peptides in lung cancer.

**DOI:** 10.1038/bjc.1982.38

**Published:** 1982-02

**Authors:** J. G. Ratcliffe, J. Podmore, B. H. Stack, W. G. Spilg, C. Gropp

## Abstract

The prevalence of high levels of circulating ACTH-like immunoactivity was determined in 134 patients with lung cancer, using reference ranges from 52 age- and sex-matched patients with non-malignant lung disease. Two studies used ACTH radioimmunoassays with different specificities. Study A used an unextracted plasma or serum assay for total ACTH immunoactivity. High serum ACTH levels occurred in 24% of patients with small-cell carcinoma and 3% of patients with non-small-cell cancer. In patients with small-cell carcinoma, levels were high in 12% with limited disease and 32% with extensive disease. Study B used an ACTH assay after plasma extraction by porous glass, which measured mainly regular 1-39 ACTH. Here no lung-cancer patient had levels above the reference range, suggesting that the high levels in Study A may be due to plasma ACTH components which are poorly extracted by porous glass. It is concluded that high circulating ACTH immunoactivity occurs in a minority of patients with lung cancer, particularly those with extensive small-cell carcinoma. Indirect evidence suggests that the high ACTH levels detected with assays for total ACTH are due to molecular forms other than 1-39 ACTH, probably high-mol.-wt species.


*Br. J. Cancer* (1982) **45**, 230

### CIRCULATING ACTH AND RELATED PEPTIDES IN LUNG CANCER

J. G. RATCLIFFE*[1], J. PODMORE*, B. H. R. STACK†, W. G. S. SPILG‡ AND C. GROPP§

*From the *Department of Biochemistry, Royal Infirmary, †Department of Respiratory Medicine, Knightswood Hospital and Western Infirmary, ‡Department of Pathology, Victoria Infirmary, Glasgow, and the §Department of Medicine, University of Marburg, Marburg, West Germany*

Received 17 July 1981 Accepted 9 October 1981

**Summary.—The prevalence of high levels of circulating ACTH-like immunoactivity was determined in 134 patients with lung cancer, using reference ranges from 52 age- and sex-matched patients with non-malignant lung disease. Two studies used ACTH radioimmunoassays with different specificities. Study A used an unextracted plasma or serum assay for total ACTH immunoactivity. High serum ACTH levels occurred in 24% of patients with small-cell carcinoma and 3% of patients with non-small-cell cancer. In patients with small-cell carcinoma, levels were high in 12% with limited disease and 32% with extensive disease.**

**Study B used an ACTH assay after plasma extraction by porous glass, which measured mainly regular 1-39 ACTH. Here no lung-cancer patient had levels above the reference range, suggesting that the high levels in Study A may be due to plasma ACTH components which are poorly extracted by porous glass.**

**It is concluded that high circulating ACTH immunoactivity occurs in a minority of patients with lung cancer, particularly those with extensive small-cell carcinoma. Indirect evidence suggests that the high ACTH levels detected with assays for total ACTH are due to molecular forms other than 1-39 ACTH, probably high-mol.-wt species.**

THERE IS NOW considerable evidence that production of ACTH and related peptides occurs commonly in lung cancer, particularly the small-cell variety, though the overt ectopic ACTH syndrome is much less prevalent (Ratcliffe & Podmore, 1980). Several authors have assessed the prevalence of circulating ACTH levels in lung-cancer patients, in an attempt to evaluate the potential clinical value of the ACTH assay as a tumour marker in diagnosis, prognosis and monitoring of lung cancer. No consensus has emerged from these studies: some authors have described high plasma ACTH levels in most patients with lung cancer of all histological types, when compared to levels in normal controls (Ayvazian *et al.*, 1975: Wolfsen & Odell, 1979). High levels were also found in up to one-third of patients with non-malignant lung disease (Gewirtz & Yalow, 1974). In contrast, others have reported high circulating ACTH levels in only a minority of lung-cancer patients, most of whom had small-cell tumours (Gropp *et al.*, 1980). However, these studies differed in several potentially important ways, including patient selection, stage of disease and tumour histology, type of sample, assay specificity and reference groups.

We have therefore attempted to define more clearly the prevalence of raised circulating ACTH-like immunoactivity in lung cancer, by comparing levels in patients with lung cancer of defined histology and stage with those in an age- and sex-matched reference group with

[1] University Department of Chemical Pathology, Hope Hospital, Salford M6 8HD.

non-malignant lung disease. Two types of radioimmunoassay have been used: an assay for "total" ACTH immunoactivity in plasma or serum without prior extraction of the hormone ("unextracted" assay) and assays for ACTH, $\beta$ - melanocyte - stimulating hormone ($\beta$MSH) and lipotrophic hormone (LPH) immunoactivities after hormone extraction from plasma ("extracted" assays).

### METHODS

*Patients studied* (Table I).—One hundred and thirty-four patients with lung cancer attending the outpatient department or admitted to hospitals in Glasgow, U.K., and Marburg, F.R.G., during 1978 and 1979 were studied. The diagnosis of lung cancer was confirmed by bronchial or pleural biopsy, biopsy of enlarged lymph nodes or other metastases, sputum cytology, or retrospectively at necropsy. Patients in whom the tumour was confined to the hemithorax, ipsilateral, mediastinal and cervical lymph nodes were considered to have localized disease. Tumour spread beyond these limits was described as extensive. Tumours were classified histologically as small-cell and non-small-cell according to the WHO classification, by pathologists in Glassgow and Marburg who were unaware of the clinical details and laboratory findings.

The reference group comprised 52 patients attending a Glasgow hospital with non-malignant pulmonary disease who were matched for age and sex with the Glasgow lung-cancer patients. None of the patients or controls was taking corticosteroid or other drugs known to affect ACTH secretion, and none had overt Cushing's syndrome. Fifty-two per cent of the control patients had been regular cigarette smokers within 1 year of the investigation.

*Blood samples.*—Ten-ml samples of venous blood were taken between 09:00 and 10:00 h into heparinized and/or plain tubes. The heparinized blood was immediately centrifuged and the plain blood was allowed to clot at room temperature. The plasma or serum was snap-frozen in dry ice, and stored at $-20$°C until assayed.

*Assays.*—Two studies were performed using different radioimmunoassays (RIA):

*Study A: Unextracted double-antibody RIA for "total" ACTH immunoactivity.*—Ninety-three sera and 44 plasma samples from 113 patients with lung cancer were assayed. Both serum and plasma samples were assayed in 24 of these patients. In addition, assays were performed on serum and plasma samples from each of 30 patients with non-malignant lung disease. This assay used human 1-39 ACTH for iodination and standardization (MRC 74/555) and an antiserum raised in rabbits against human 1-39 ACTH (kindly supplied by Dr L. Husager, Medi-Lab, Denmark). When compared to human 1-39 ACTH, this antiserum cross-reacted 98% with 1-24 ACTH, but showed no significant cross-reaction with human 18-39 ACTH, human $\beta$ and $\gamma$LPH, human $\beta$MSH, $\beta$-endorphin and leu- and met-enkephalins. The ACTH assay was performed as follows: 200 $\mu$l serum, plasma or standard solution was incubated overnight at 4°C with 200 $\mu$l saline/albumin diluent, 100 $\mu$l antiserum (final dilution 1:48,000 in EDTA containing phosphate buffer) and 100 $\mu$l $^{125}$I-ACTH ($\sim$25 pg). Carrier normal rabbit serum and donkey anti-rabbit serum (100 $\mu$l each) were added and incubated overnight at 4°C. After

TABLE I.—*Clinical details of patients studied*

| | Study A | | | | Study B | | | |
|---|---|---|---|---|---|---|---|---|
| | *Lung cancer* | | *Controls* | | *Lung cancer* | | *Controls* | |
| Total | 113 | | 30 | | 21 | | 22 | |
| Males (No.) | 99 | | 23 | | 18 | | 18 | |
| Mean age (years) | 63 | | 62 | | 66 | | 65 | |
| Age range | 46–78 | | 45–85 | | 53–82 | | 51–78 | |
| Diagnosis | Small-cell | 73 | Chronic bronchitis | 17 | Small-cell | 7 | Chronic bronchitis | 14 |
| | Non-small-cell | 40 | Bronchopulmonary infection | 7 | Non-small-cell | 14 | Bronchopulmonary infection | 3 |
| | | | Asthma | 3 | | | Asthma | 1 |
| | | | Miscellaneous | 3 | | | Miscellaneous | 4 |

centrifugation and aspiration of the supernatant, the bound fraction was counted. The assay was validated by comparison of ACTH values obtained with the unextracted assay at a plasma dilution of 1:4 with those found with the N-terminal ACTH assay on extracted plasma described below. A good correlation between methods was found in 61 paired samples from normal subjects and patients with a wide range of non-malignant conditions associated with abnormal ACTH status (unextracted ACTH value = 0·99 × extracted ACTH value + 7·33, $r = 0{\cdot}960$). The limit of detection of the unextracted assay was 10–20 ng/l, and interassay coefficient of variation was 16%.

*Study B: Extracted RIA for ACTH, "βMSH", and LPH immunoactivity.*—Plasma samples from 21 patients with lung cancer and from 22 patients with non-malignant lung disease were assayed for ACTH, "βMSH" and LPH immunoactivity after prior extraction. For each hormone, a 5ml plasma aliquot was extracted with porous glass by the method of Ratcliffe & Edwards (1971). This method selects against high-mol-wt ACTH components and C-terminal ACTH fragments so that the extracted ACTH assay is relatively specific for 1-39 ACTH.

The ACTH RIA was as described by Rees *et al.* (1971) using iodinated human 1-39 ACTH as tracer, an antiserum directed towards the biologically active 1-24 region of the molecule, and standardized against natural human ACTH (MRC 74/555). The limit of detection was 10 ng/l.

"βMSH" immunoactivity was assayed by the method of Gray & Ratcliffe (1979) standardized with synthetic human βMSH (Ciba) using an antiserum which cross-reacts equally on a molar basis with human βMSH, βLPH and γLPH. There was no cross-reaction with ACTH, its fragments or endorphin or enkephalins. The limit of detection was 10 ng/l.

LPH immunoactivity was assayed by the method of Podmore (1979) using iodinated human βLPH and an antiserum which cross-reacts equally on a molar basis with human β and γLPH. There was no cross-reaction with human βMSH, ACTH or its fragments, β-endorphin or enkephalins. The assay was standardized against purified human βLPH (Dr P. J. Lowry). The limit of detection was 80 ng/l.

*Statistical analysis* of grouped data was by the Mann Whitney U test. Where an individual value was undetectable a figure of half the formal limit of detection was assumed.

### RESULTS

### *ACTH values in plasma compared to serum*

In order to determine whether ACTH levels differ in plasma and serum, both plasma and serum samples were taken at the same time from 30 control patients and the values compared. For plasma samples, the median value was between 20 and 30 ng/l and the absolute range was < 20–73 ng/l. For serum samples, the

TABLE II.—*Prevalence of high total-ACTH levels in serum and plasma in patients with lung cancer*

| | Plasma | | Serum | |
|---|---|---|---|---|
| Lung cancer | Total No. | No. > control range (< 20–73 ng/l) (%) | Total No. | No. > control range (< 20–50 ng/l) (%) |
| Small-cell | | | | |
| Limited | 6 | 0 (0) | 25 | 3 (12)* |
| Extensive | 12 | 3 (25) | 38 | 12 (32)† |
| All cases | 18 | 3 (17) | 63 | 15 (24)† |
| Non-small-cell | | | | |
| Limited | 20 | 0 (0) | 20 | 0 (0) |
| Extensive | 6 | 0 (0) | 10 | 1 (10) |
| All cases | 26 | 0 (0) | 30 | 1 (3) |
| All lung-cancer cases | 44 | 3 (7) | 93 | 16 (17)† |

* $P < 0{\cdot}01$ *vs* controls.

† $P < 0{\cdot}001$ *vs* controls.

median value was < 20 ng/l and the absolute range was < 20–50 ng/l.

It is concluded that plasma ACTH levels are slightly higher than serum values. In view of this, the subsequent prevalence data in lung cancer patients in Study A are related to the reference range obtained in the appropriate type of sample: *e.g.*, unextracted serum ACTH levels in lung cancer patients are related to the control range in unextracted serum.

*Study A (total ACTH immunoactivity)*

Table II summarizes the prevalence of high total ACTH levels in plasma or serum expressed in relation to the appropriate plasma or serum reference range. The patterns are similar for plasma and serum, though the smaller number of plasma samples probably makes those figures less reliable for the small cell group. Serum ACTH levels were significantly higher in patients with either limited or extensive small-cell cancer than in controls or non-small-cell cancer. Likewise the prevalence of high serum ACTH levels is much greater in patients with small-cell than non-small-cell cancer (24% *vs* 3%). Only 1 patient with non-small-cell cancer had a high serum ACTH level (55 ng/l). In the small-cell group, high serum levels were related to stage of disease, being ~2·5-fold more frequent in patients with extensive disease. However, even in patients with high ACTH levels and small-cell cancer the degree of abnormality was modest. Six patients had levels > 100 ng/l, and only 1 had a level > 200 ng/l (250 ng/l).

*Study B (extracted ACTH, βMSH and LPH immunoactivity*

Table III summarizes the prevalence of elevated ACTH, βMSH and LPH levels in extracted plasma. In contrast to Study A, the levels are uniformly within the reference range, except for a single patient with non-small-cell cancer with a marginally high LPH (356 ng/l). Although the number of patients is small, particularly those with small-cell carcinoma, these data confirm the low prevalence of high levels of ACTH and related peptides in lung cancer, and suggest that the prevalence with the extracted ACTH assay is much less than with the unextracted assay.

### DISCUSSION

Our observations suggest that the prevalence of high levels of ACTH immunoactivity in patients with lung cancer is relatively low, is mainly associated with extensive small-cell cancer, and is apparently related to the specificity of the ACTH assay. The prevalence is similar to that reported by Gropp *et al.* (1980), Hansen *et al.* (1980*a*) and Torstensson *et al.* (1980) but much lower than described by Ayvazian *et al.* (1975), Yalow *et al.* (1979) and Wolfsen & Odell (1979) (Table IV). We have attempted to analyse some of the factors which may account for these discrepancies.

*Type and time of sampling*

Comparison of unextracted ACTH levels in plasma and serum samples (Table II) shows that levels are lower in serum than

TABLE III.—*Prevalence of high plasma levels of ACTH, βMSH and LPH using extraction methods*

| | ACTH (ng/l) | | | βMSH (ng/l) | | | LPH (ng/l) | | |
|---|---|---|---|---|---|---|---|---|---|
| Lung cancer | Range | No. | No. > control range (< 10–73) | Range | No. | No. > control range (14–62) | Range | No. | No. > control range (< 80–341) |
| Small-cell | < 10–34 | 7 | 0 | 13–51 | 7 | 0 | < 80–256 | 7 | 0 |
| Non-small-cell | < 10–35 | 14 | 0 | 16–49 | 14 | 0 | < 80–356 | 14 | 1 |
| All cases | < 10–35 | 21 | 0 | 13–51 | 21 | 0 | < 80–356 | 21 | 1 |

TABLE IV.—*Prevalence of elevated ACTH concentrations in plasma and serum of lung cancer patients before treatment*

| Authors | Patients | Sample and method | Reference value (ng/l) | Derivation of reference value | Percentage prevalence of patients with values above reference value: Small-cell | Non-small-cell | All patients |
|---|---|---|---|---|---|---|---|
| Gropp *et al.*, 1980 | Unselected | Plasma and serum unextracted | 80 | Age- and sex-matched normal controls | 30 | 10 | 19 |
| Yalow *et al.*, 1979 | Surgical non-small-cell | Plasma unextracted | 150 | Normal control range. Sampled, p.m. | — | 72 | 72 |
| Ayvazian *et al.*, 1975 | Unselected | Plasma unextracted | 150 | Normal control range. Sampled, p.m. | 100 | 72 | 88 |
| Hansen *et al.* 1980*a* | Unselected small-cell | Plasma unextracted | 76 | Mean + 2 s.d. of laboratory controls | 29 | — | 29 |
| Wolfsen & Odell, 1979 | Unselected radiographic abnormality | Plasma Extracted | 107 | Mean + 2 s.d. of normals and patients with minor illness. Sampled, a.m. | — | — | 74 |
| Torstensson *et al.*, 1980 | Unselected | Plasma unextracted | 216 | Upper limit of absolute range in benign pulmonary disease. Sampled, p.m. | — | — | 17 |

plasma, possibly due to loss of immunoactivity during clotting, since it is well recognized that endogenous ACTH and exogenous human 1-39 ACTH are unstable in whole blood (Besser *et al.*, 1971). However, comparison of plasma or serum levels in lung-cancer patients with their counterparts in control patients gave similar prevalence figures, suggesting that differences in sample handling cannot account for large variations in prevalence.

Our samples were taken in the morning, when higher ACTH levels may be expected. Others have reported a high or low prevalence irrespective of time of sampling (Table IV).

*Definition of reference range*

Most previous studies have compared values in lung-cancer patients with those in normal subjects (either as absolute reference ranges or 95% confidence limits). We consider that age- and sex-matched patients with non-malignant lung disease and comparable smoking histories form a more appropriate reference group, since lung cancer often occurs on a background of other lung pathology. Immunoactive ACTH has been described in bronchial epithelium of a smoking dog with atypical premalignant changes (Gewirtz & Yalow, 1974). Such changes are described in heavy smokers who died of other disease as well in the lungs of lung-cancer patients (Auerbach *et al.*, 1961). It is thus possible that circulating ACTH may be increased by concomitant epithelial changes as well as by tumour secretion. In addition, neuroendocrine activation in disease-induced stress may enhance pituitary ACTH secretion. In support of this, Torstensson *et al.* (1980) found higher ACTH levels in patients with non-malignant lung disease than in normal subjects.

However, the selection of control patients cannot explain the different prevalences, since higher or lower figures are reported when normal subjects are used as controls. Although the low prevalence reported by Torstensson *et al.* (1980) may be in part related to the high control reference range, our reference range was similar to what we have found in healthy subjects. It is noteworthy that the highest prevalence figures were in studies that also reported much higher reference values than the present study. This implies differences in assay standardization and specificity (*vide infra*).

*Nature and extent of tumour*

The ectopic ACTH syndrome is most commonly due to small-cell carcinoma of lung (Broder, 1979). Other histological types of lung cancer rarely cause the syndrome. The present Study A, and that of Gropp *et al.* (1980), find a greater prevalence of raised ACTH levels in small-cell carcinoma, whereas Yalow *et al.* (1979) report a high prevalence in non-small-cell cancer. The latter is difficult to reconcile with a tumour origin of ACTH, since tumour-tissue concentrations are usually very low or negative for ACTH and related peptides in this histological type. It therefore seems unlikely that differences in tumour histology can account for the variation in prevalence. Similarly, differences in degree of tumour spread does not readily account for the discrepancies. Yalow *et al.* (1979) studied surgical cases with presumably Stage I and II tumours, and Wolfsen & Odell (1979) investigated patients with peripheral coin lesions and other localized radiographic abnormalities. In contrast, our series contained patients with extensive disease in whom circulating ACTH levels might be expected to be higher.

*Assay specificity*

A major problem in comparing previous reports is evaluation of assay specificity. Tumour and plasma ACTH from patients with ectopic ACTH syndrome, is heterogeneous, with a high but variable proportion of high-mol.-wt species and fragments (Ratter *et al.*, 1980; Pullan *et al.*, 1980). Assay specificity with respect to these components is uniformly poorly defined, since purified materials are not available for formal cross-reaction studies. Most authors have employed assays which detect the N-terminal portion of ACTH and some, including our unextracted assay cross-react to an unknown extent with high-mol.-wt species.

Extraction by porous glass increases the specificity towards 1-39 ACTH and our finding of a reduced prevalence with the extracted assay confirms the report of Gewirtz & Yalow (1974) that high-mol.-wt ACTH is a major component of ACTH-producing lung tumours. The low prevalence reported with a radioreceptor assay which measures predominantly 1-39 ACTH (Wolfsen & Odell 1979) also supports this contention. Against this, these authors also reported a high prevalence using their extracted ACTH assay which might be expected to select against high-mol.-wt forms of ACTH. Clearly, further work is required using assays of defined specificities to all the ACTH-like components in lung-cancer plasma, as assay specificity seems likely to be a major factor in accounting for these discrepancies.

In conclusion, our results show that high serum ACTH immunoactivity occurs in less than 20% of patients with lung cancer, but is commoner in extensive small-cell cancer. ACTH assays thus appear to have no value in screening or diagnosis of lung cancer. However, in patients with high ACTH levels, serial measurements could be useful in assessing response to treatment and in detecting recurrence (Hansen *et al.*, 1980*b*). Further studies should devote attention to the type of sample, selection of reference group, tumour histology and spread. However, we consider that definition of the nature of circulating ACTH in lung cancer, with development of assays specific for the tumour-related components, is a vital precondition for definitive evaluation of ACTH levels in lung cancer.

This work was supported by a grant from the Scottish Hospitals Endowment Research Trust to J. G. Ratcliffe. We are grateful to Mrs A. McKinnon for secretarial help.

### REFERENCES

AUERBACH, O., STOUT, A. P., HAMMOND, E. C. & GARFINKEL, L. (1961) Changes in bronchial epithelium in relation to cigarette smoking and in relation to lung cancer. *N. Engl. J. Med.*, **265**, 253.

AYVAZIAN, L. F., SCHNEIDER, B., GEWIRTZ, G. & YALOW, R. S. (1975) Ectopic production of big ACTH in carcinoma of the lung. *Am. Rev. Resp. Dis.*, **111**, 279.

BESSER, G. M., ORTH, D. N., NICHOLSON, W. E., BYNY, R. L., ABE, K. & WOODHAM, J. P' (1971) Dissociation of the disappearance of bioactive and radioimmunoactive ACTH from plasma in man. *J. Clin. Endocrinol. Metab.*, **32**, 595.

BRODER, L. (1979) Hormone production by bronchogenic carcinoma: a review. *Pathobiol. A.*, **9**, 205.

GEWIRTZ, G. & YALOW, R. S. (1974) Ectopic ACTH production in carcinoma of the lung. *J. Clin. Invest.*, **53**, 1022.

GRAY, C. E. & RATCLIFFE, J. G. (1979) Clinical evaluation of a radioimmunoassay for βMSH related peptides. *Clin. Endocrinol.*, **10**, 163.

GROPP, C., HAVEMANN, K. & SCHEUER, A. (1980) Ectopic hormones in lung cancer patients at diagnosis and during therapy. *Cancer*, **46**, 347.

HANSEN, M., HANSEN, H. H., HIRSCH, F. R. & 5 others (1980*a*) Hormonal polypeptides and amine metabolites in small cell carcinoma of the lung with special reference to stage and subtypes. *Cancer*, **45**, 1432.

HANSEN, M., HAMMER, M. & HUMMER, L. (1980*b*) ACTH, ADH and calcitonin concentrations as markers of response and relapse in small cell carcinoma of the lung. *Cancer*, **46**, 2062.

PODMORE, J. (1979) *Inappropriate Hormone Production by Human Tumours*. University of Glasgow: PhD Thesis.

PULLAN, P. T., CLEMENT-JONES, V., CORDER, R., LOWRY, P. J., BESSER, G. H. & REES, L. H. (1980) ACTH, LPH and related peptides in the ectopic ACTH syndrome. *Clin. Endocrinol.*, **13**, 437.

RATCLIFFE, J. G. & EDWARDS, C. R. W. (1971) Extraction of ACTH and AUP from human plasma by porous glass. In *Radioimmunoassay Methods* (Eds Kirkham & Hunter). Edinburgh: Churchill Livingstone. p. 502.

RATCLIFFE, J. G. & PODMORE, J. (1980) Ectopic hormones. In *Cancer: Assessment and Monitoring* (Eds Symington *et al.*). Edinburgh: Churchill Livingstone. p. 324.

RATTER, S. J., LOWRY, P. J., BESSER, G. M. & REES, L. H. (1980) Chromatographic characterization of adrenocorticotrophin in human plasma. *J. Endocrinol.* **85**, 359.

REES, L. H., COOK, D., KENDALL, J. W. & 4 others (1971) A radioimmunoassay for rat plasma ACTH. *Endocrinology*, **89**, 259.

TORSTENSSON, S., THOREN, M. & HALL, K. (1980) Plasma ACTH in patients with bronchogenic carcinoma. *Acta Med. Scand.*, **207**, 353.

WOLFSEN, A. R. & ODELL, W. D. (1979) Pro ACTH: use for early detection of lung cancer. *Am. J. Med.*, **66**, 765.

YALOW, R. S., EASTRIDGE, C. E., HIGGINS, G. & WOLF, J. (1979) Plasma and tumour ACTH in carcinoma of the lung. *Cancer*, **44**, 1789.